# Generalized Anxiety Disorder Associated With Gastroesophageal Reflux Disease Among the Saudi Population

**DOI:** 10.7759/cureus.50175

**Published:** 2023-12-08

**Authors:** Razana Baeisa, Duaa M Bakhshwin, Emad Aljahdli, Wid Kattan, Wafaa H Alhashmi, Eilaf Metwalli, Renad A Almutiry, Alya Alrehaili, Asalah A Alammari, Manar Alharbi

**Affiliations:** 1 Faculty of Medicine, King Abdulaziz University, Jeddah, SAU; 2 Department of Clinical Pharmacology, King Abdulaziz University, Jeddah, SAU; 3 Department of Gastrointestinal Oncology, King Abdulaziz University Hospital, Jeddah, SAU; 4 Department of Psychiatry, Department of Medicine, King Abdulaziz University, Jeddah, SAU

**Keywords:** prevalence, cross-sectional studies, gerdq, saudi arabia, gastroesophageal reflux, general anxiety disorder-7

## Abstract

Objectives

In Saudi Arabia, the prevalence of generalized anxiety disorder (GAD) was reported to be 29%. As a result, our goal was to examine the association between GAD and gastroesophageal reflux disease (GERD) within the general Saudi Arabian population, as well as to access the risk factors for GAD in order to gain a better understanding.

Method

This cross-sectional study involved 4,224 participants who completed a questionnaire. Anxiety was assessed using the General Anxiety Disorder-7 (GAD-7) scale, and the GerdQ tool was used to evaluate GERD.

Result

The prevalence of anxiety among participants was 29% at cutoff 10, with 73% of anxiety-positive participants being female and only 26.9% being male. Furthermore, the associations between anxiety and GERD were significant as 31.4% of participants with anxiety had GERD, compared to 15.0% of those without anxiety.

Conclusion

In our finding, there was a significant association between anxiety and GERD among the general Saudi population. In terms of anxiety risk factors, female, younger age, social status, body mass index, eating fried food, caffeinated drinks, diabetes miletus, high blood cholesterol, NSAID use, antidepressants, and anti-anxiety medication were found to have a significant association.

## Introduction

Generalized anxiety disorder (GAD) is a psychological disorder associated with uncontrolled worry about everything, and people with GAD are often overwhelmed by something terrible in terms of money, health, family, work, and other issues [1]. Additionally, they usually experience physical symptoms, including restlessness, worry, difficulty concentrating, difficulty sleeping, lightheadedness, and increased heart rate [2]. GAD can happen to anyone at any age. However, the most significant factors were younger age and female sex [1]. Furthermore, from an economic point of view, GAD patients spent a total of 2867€ more per year compared to non-GAD patients, divided into direct and indirect costs, including hospital visits to psychologists and psychiatrists and due to associated comorbidities, and indirect costs involved with disability in work engagement and absenteeism [3].

In terms of prevalence, a study conducted in the United States in 2021 reported an anxiety prevalence of 21.4% [4]. While in Asia, a study conducted in the northern province of Sri Lanka in 2019 reported that anxiety prevalence was 46.7% [5]. According to the Middle East, a study in the United Arab Emirates in 2019 reported the prevalence of anxiety was 56% [6].

Locally in Saudi Arabia, a cross-sectional study was conducted among the general population using the General Anxiety Disorder-7 (GAD-7) questionnaire in 2021, which found the prevalence of moderate and severe anxiety was reported at 29% [1].

Psychological variables, such as anxiety and stress, can affect normal gastrointestinal (GI) function and contribute to various GI disorders, including gastroesophageal reflux disease (GERD) [7]. GERD occurs when a weak lower esophageal sphincter fails to close properly, which can lead to the backflow of the acidic contents of the stomach, irritating the epithelial lining of the esophagus [8]. History and physical examination are both primarily used to diagnose GERD, occasionally when there is an atypical presentation, or to assess the severity and complications of GERD. There are several tools that can help, including ambulatory pH monitoring, manometry, and endoscopy [9].

The prevalence of GERD counts for about 10-20% in the Western population [10], while, in the Middle East, the estimated range of GERD prevalence was about 8.7-33.1% [11]. Furthermore, in Saudi Arabia, a study conducted in 2018 reported a prevalence of 28.7%, and in another study conducted in Riyadh province in 2014, a prevalence accounted for 45.4%. Both studies used GerdQ [12,13].

A study conducted in 2021 reported an increased risk of anxiety, depression, and sleep disorders in patients with GERD in India [14]. In Korea, a cross-sectional study of 19,099 participants reported higher anxiety levels among patients with GERD in 2018 [15]. Additionally, Iran’s study in 2017 found that anxiety and depression are significant mental health factors in the development of GERD [16]. Another study conducted in India in 2017 found that GERD adversely affects quality of life and increases the risk for anxiety and depression, creating a vicious cycle [17], while a prospective study conducted in China in 2015 reported that anxiety may be a significant factor in the development of GERD [8]. In Saudi Arabia, there is a lack of studies observing the association between GAD and GERD, and insufficient studies with large sample sizes to assess the prevalence of GAD and GERD in the general population. This study aimed to evaluate the association between GAD and GERD in the general Saudi Arabian population.

## Materials and methods

This cross-sectional study was conducted among the general population of Saudi Arabia from January 2021 to March 2023 after approval by the research ethics committee at King Abdulaziz University Hospital on the 26th of January 2022, with reference number HA-02-J-008.

Our inclusion criteria were Saudi Arabian residents who had access to social media, were aged 18 years or older, and could read Arabic or English. A total of 4,714 responses were received from all the provinces of Saudi Arabia. The responses were narrowed down to 4,224 participants, excluding those with esophageal cancer, gastroparesis, peptic ulcer disease, gastrectomy, pregnant women, and those who refused to complete the questionnaire.

All study participants completed a self-administered questionnaire designed through Google Forms after receiving the link through social media platforms, such as WhatsApp and Twitter. The questionnaire consisted of three sections available in Arabic and English. The first part included sociodemographic data, such as sex, age, height, weight, marital status, educational level, residency, medical conditions, current medication use, smoking status, and dietary habits.

The second part was the validated GAD-7, which is a screening tool for anxiety disorders that asks individuals how frequently they have experienced anxiety symptoms during the past two weeks. The validated Arabic version was used with permission from AlHadi et al. [18]. The GAD-7 scale comprises seven main components: (1) feeling nervous, anxious, or on edge; (2) not being able to stop or control worrying; (3) worrying excessively about various things; (4) difficulty relaxing; (5) being so restless that it is challenging to sit still; (6) becoming easily annoyed or irritable; and (7) feels that something terrible might happen. Every component is scored from 0 to 3, which represent as follows: 0 for “not at all,” 1 for “several days,” 2 for “more than half the days,” and 3 for “almost every day,” respectively, and total score arrange from 0 to 21 points. As cutoff points, 5, 10, and 15 total scores were recorded for mild, moderate, and extreme anxiety, respectively.

GAD-7 showed a sensitivity of 89% and a specificity of 82% to find anxiety symptoms in GAD. We used a cutoff value for anxiety with a score ≥10, reflecting the optimal balance between sensitivity and specificity [19].

The third part of the questionnaire was used for GERD evaluation using the Arabic version of the GerdQ tool, with permission from Almadi et al. [12]. It scores the frequency of four positive predictors of GERD (heartburn, regurgitation, sleep disturbance due to reflux symptoms, or use of over-the-counter medications for reflux symptoms on a four-graded Likert scale (0-3) and two negative predictors of GERD (epigastric pain and nausea) on a reversed Likert scale (3-0), and participants were asked to recall the previous seven days. The scores range from 0 to 18 [13]. Our cutoff value for GerdQ was ≥ 9, which provided the optimal balance between sensitivity (66%) and specificity (64%) [20]. The study's primary outcome was the association between anxiety and GERD in the general Saudi population. Additionally, we assessed the risk factors for GAD and its prevalence as a secondary outcome.

Statistical analysis

The data were entered using Microsoft Excel 2021 and Statistical Product and Service Solutions (SPSS, version 21) (IBM SPSS Statistics for Windows, Armonk, NY). In the SPSS program, a one-way analysis of variance (ANOVA) was used to compare the mean continuous variables, and the chi-square test was used for categorical variables. Screening for critical clinical parameters using univariate logistic regression variables with a P-value of P<0.05 was considered statistically significant and included in multiple multinomial logistic regression.

## Results

The primary purpose of this study was to examine the association between anxiety and GERD in the general Saudi population, and the secondary finding was the prevalence of GAD and related risk factors.

Characteristics and demographics of the study subjects

The study included 4,224 participants from all over the Kingdom of Saudi Arabia. A total of 2,572 (60.9%) were females, and only 1,652 (39.1%) were males, with the mean age of the participants of 31.12±11.008 standard deviation (SD). Most participants had average weight according to their BMI, and 70.1 % of the study sample had a university degree.

Anxiety and sociodemographic factors

**Figure 1:**
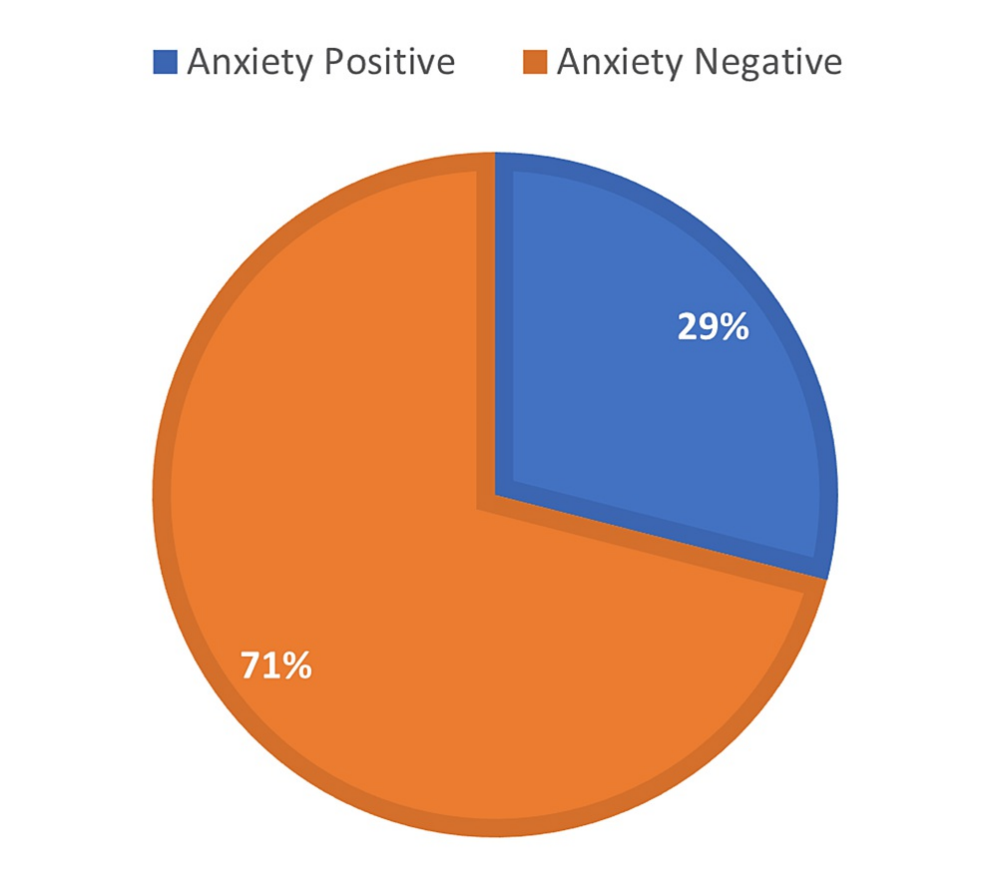
Anxiety prevalence

A total of 4,224 participants completed the GAD-7 questionnaire. Anxiety prevalence has been found at 29%, at a cutoff ≥10 (Figure 1). Moreover, the participant among the anxiety positive group 73% of them were females, and only 26.9% were males. Sociodemographic participants’ anxiety and negative anxiety are presented in Table 1. There was a significant difference in gender, age, social status, and BMI. Moreover, the correlation between age and anxiety score was -.181, indicating an indirect relationship, which means increasing the GAD-7 score with decreasing participant age.

**Table 1:** Sociodemographic of participants: anxiety positive and anxiety negative p-values were derived from the chi-square test; significance was set at p<0.05. GERD, gastroesophageal reflux disease; BMI, body mass index

Variables	Anxiety Negative		Anxiety Positive		p-value
	n	%	n	%	
Gender					<0.001
Male	1325	44.0%	327	26.9%	
Female	1684	56.0%	888	73.1%	
Age					<0.001
18-27	1396	46.4%	750	61.7%	
28-37	694	23.1%	225	18.5%	
38-47	572	19.0%	165	13.6%	
48-57	270	9.0%	57	4.7%	
≥58	77	2.6%	18	1.5%	
Age (T-test)	32.15±11.205		28.55±10.959		<0.001
Marital status					<0.001
Single	1521	50.5%	793	65.3%	
Married	1364	45.3%	375	30.9%	
Other	124	4.1%	47	3.9%	
Education Level					<0.001
High School	517	17.2%	264	21.7%	
University	2123	70.6%	837	68.9%	
Other	369	12.3%	114	9.4%	
BMI					<0.001
Underweight	196	6.7%	150	12.6%	
Normal	1257	42.7%	521	43.9%	
Overweight	881	29.9%	300	25.3%	
Obese	402	13.7%	116	9.8%	
Extremely obese	206	7.0%	101	8.5%	
(T-Test)	25.860203±5.923438		24.978849±6.400898		0.000203

Eating habits, medical disorders, and medication associated with anxiety

Anxiety was found to have a significant relationship with chocolate consumption, caffeinated drinks, and DM. No differences were found between the groups regarding smoking status, having irritable bowel syndrome, or thyroid disorders, as detailed in Table 2.

**Table 2:** Eating habits, medical disorders, and medication relation with anxiety (n=4,224) IBS, irritable bowel syndrome; DM, diabetes mellitus; HTN, hypertension; NSAIDS, nonsteroidal anti-inflammatory drugs. p-values were derived using the chi-square test. Significant at the level of p<0.05

Variables	Anxiety Negative		Anxiety Positive		p-value
	n	%	n	%	
Smoking	701	23.3%	297	24.4%	.450
Eating Chocolate	2679	89%	1136	93.5%	<0.001
Eating fried and fatty food	2761	91.8%	1167	96%	<0.001
Arabic coffee	2021	67.2%	684	56.3%	<0.001
Energy drinks and espresso consumption	1319	43.8%	616	50.7%	<0.001
High blood cholesterol	213	7.1%	105	8.7%	0.091
IBS	459	15.3%	194	16.0%	0.607
Asthma	31	1.0%	10	0.8%	0.651
DM	48	1.6%	32	2.6%	0.034
HTN	27	0.9%	17	1.4%	0.201
Depression diagnosis					<0.001
Current	50	1.7%	95	7.8%	
Previously	91	3.0%	119	9.8%	
Anti-anxiety medications	26	0.9%	59	4.9%	<0.001
Anti-depressant medications	61	2.0%	75	6.2%	<0.001
NSAIDs	140	4.7%	92	7.6%	<0.001

GERD symptoms in patients with anxiety

The prevalence of GERD among the participants was 19.7% (n=4,224), based on the GerdQ questionnaire. Moreover, GAD and GERD have a significant association (P<0.001), Anxiety-positive participants had GERD at a rate of 31.4% (n=381), while anxiety-negative people had GERD at a rate of 15% (n=460). In addition, the percentage of GERD-positive participants increased significantly as their anxiety levels increased (Table 3).

**Table 3:** GAD and GERD association

Anxiety categories	GERD Negative		GERD Positive		p-value
	n	%	n	%	
Negative anxiety	1654	88.8	209	11.2	<0.001
Mild anxiety	905	79	241	21	
Moderate anxiety	450	69.1	201	30.9	
Severe anxiety	384	68.2	179	31.8	

**Figure 2:**
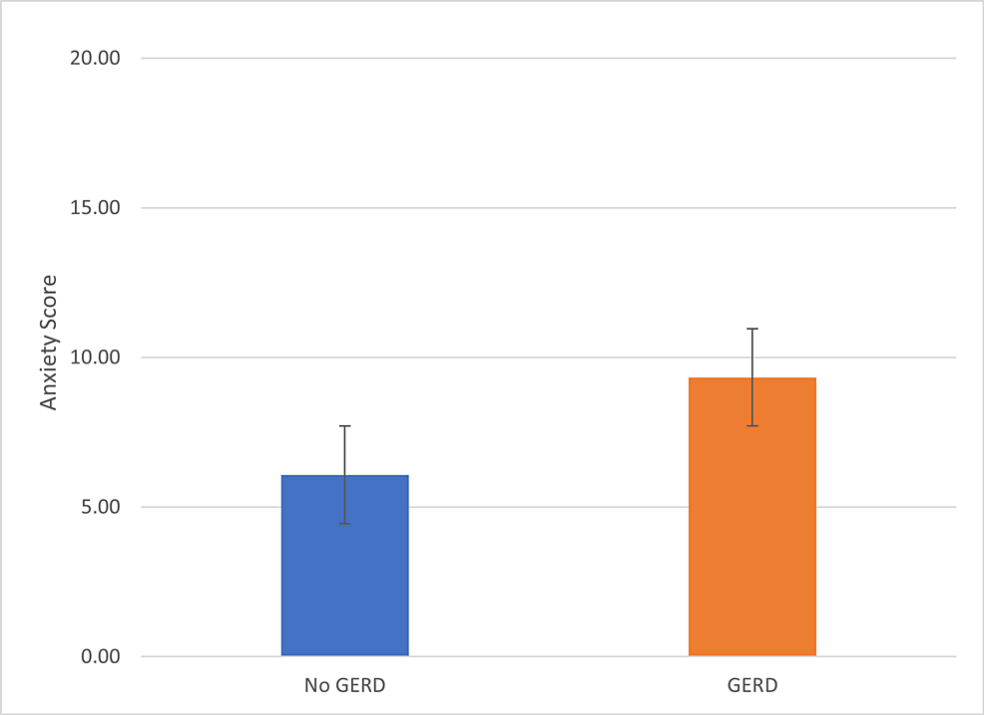
Comparison of the GAD-7 scores in GERD positive and GERD negative

Figure 2 shows the difference in anxiety scores between the participants with negative and positive GERD. Furthermore, in the anxiety-positive group, the GERD score was (7.37±2.494), while in anxiety negative, the score was (6.73±1.840). This reports a significant relation using the t-test. Considering that the correlation between the GAD-7 and GERD score was 176, there was a direct relationship between GAD and GERD. Correlation is significant at the 0.01 level (2-tailed).

Table 4 shows the logistic regression of several variables that may have an impact on the outcome; all variables were adjusted for each other.

**Table 4:** Logistic regression Dependent variable of anxiety diagnosis. All factors adjusted for others. "Reference category "first

Variables	p	OR	Confidence interval	
			Lower	Upper
Gender (Female)	0.000	2.534	2.126	3.021
Age	0.000	0.977	0.967	0.988
BMI	0.541	0.996	0.983	1.009
Social status: Single0	0.032			
Married	0.009	0.749	0.604	0.929
Other	0.266	0.785	0.513	1.202
Educational status: High School	0.010			
University	0.080	1.298	.970	1.736
Other	0.859	0.977	0.756	1.263
Fatty or fried food	0.009	1.597	1.123	2.270
Chocolate	0.235	1.198	.889	1.612
Smoking	0.005	1.313	1.084	1.591
Arabic coffee	0.000	0.733	0.628	0.856
Energy drink	0.018	1.249	1.039	1.501
Other types of coffee, such as espresso	0.187	.899	.768	1.053
DM	0.043	1.677	1.018	2.765
Current depression	0.000	4.626	2.971	7.203
Previous depression	0.000	3.474	2.536	4.761
NSAIDs	0.060	1.342	0.988	1.823
Anti-depression meds	0.168	0.711	0.437	1.155
Anti-anxiety meds	0.003	2.337	1.329	4.111
GERD positive	0.000	2.870	2.411	3.418

## Discussion

In this study, we aimed to observe the associations between GAD and GERD among the general population in Saudi Arabia, as well as the risk factors for GAD. A good understanding of the association between GERD and psychological factors is valuable for enhancing the management of GERD and improving the patient’s quality of life.

Sociodemographic

Prevalence and Gender of GAD

The prevalence of GAD in our study was 29%, according to the GAD-7 questionnaire, with a cutoff ≥10. A similar result has been shown by research on the general population of Saudi Arabia with a 2021 sum of moderate and severe anxiety at 29% [1]. Moreover, in this study, 73.1% of anxiety-positive participants were female, and gender is considered a significant factor in the development of GAD. A recent study in Saudi Arabia found similar results, reporting that anxiety was higher among females than males but with no significant relationship [1]. Several other studies have found that females are more affected by anxiety than males and explained these differences in prevalence as females being more vulnerable to stress and post-traumatic disorder [21,22]. This could also be due to changes in brain chemistry and hormone levels during reproductive events [23].

Age 

The results showed that participants in the age group 18-27 years represented the majority of anxiety-positive participants, accounting for 61.7%. A study conducted in Saudi Arabia also found that younger participants were more affected by anxiety than were older participants [1]. This reflects the fact that the young population faces numerous stressors affecting their anxiety levels, such as concerns about academic achievement, the need for a job, the preparation for new families, and the construction of their future.

Eating the happiest - fatty and fried food

We found a significant relationship between the consumption of fatty or fried food and anxiety. A cross-sectional study conducted in Iran found that adherence to a healthy diet is associated with lower rates of anxiety [24]. This could be due to the consumption of fatty or fried food, which may lead to an increase in body weight and anxiety, which may be more common in obese or overweight people than in normal-weight individuals [25]. Furthermore, individuals with anxiety disorders tend to eat fatty and fried food for temporary pleasure to mask their anxiety.

Medical condition

Diabetes Mellitus 

The present study revealed a significant association between anxiety and diabetes mellitus. Furthermore, in a cross-sectional study in Qatar, face-to-face interviews were conducted with patients with DM and controls using a questionnaire that captured the sociodemographic characteristics of subjects and the short version of the Depression Anxiety Stress Scales (DASS)-21 questionnaire, revealing that a significantly larger proportion of patients with DM had severe anxiety scores [26]. A possible explanation for this significant relationship is that patients with diabetes are always under a medical burden due to the complications of their disease.

Anxiety Association with GERD

There is a significant association between anxiety and GERD. Furthermore, the number of GERD-positive participants increased as their anxiety levels increased.

In light of this association, many studies have been conducted to investigate this relationship; one study conducted in Pakistan in 2021 reported a significant relationship between GERD and anxiety [7]. Additionally, an Australian study in 2017 revealed a strong independent association between GERD and anxiety [27], and another 2015 study in China had a similar finding as well [8]. As an explanation for this association, the hypothesis of anxiety can be a result of GERD-induced anxiety that chronic reflux may lead to chronic fatigue and sleeping difficulties, affecting the patient's social interaction, and leading to anxiety [7]. Another explanation for the GI symptoms is that they may be manifestations of anxiety disorders [28]. However, it is not clear whether anxiety induces GERD or vice versa.

Limitation

This study was conducted in a large population in Saudi Arabia. The main limitation of this study was its cross-sectional design, which reflects the association, not the causation, between anxiety and GERD. Second, the participants answered questions that could sometimes be untruthful or biased. Lastly, although a validated questionnaire was used to evaluate these factors, it was designed for screening rather than diagnosis, which may overstate the truth.

## Conclusions

In conclusion, anxiety was significantly and directly correlated with GERD, and increasing anxiety levels increased the risk of developing GERD. Regarding anxiety risk factors, a significant association was found between female sex, younger age, social status, higher BMI, consumption of fried food, caffeinated drinks, DM, high blood cholesterol, NSAID use, antidepressant use, and anti-anxiety medications. There were no significant associations between anxiety and smoking, irritable bowel syndrome, hypertension (HTN), asthma, or thyroid disorders. Further studies are required to investigate the causality between anxiety and GERD.

## Appendices

**Table 5:** Author contributions

Authors contributions	Author name
Conceptualization, Methodology, Software, validation, formal analysis, investigation, resources, data curation, writing - original draft, writing - review & editing.	Razana Saleh Baeisa
Conceptualization, methodology supervision, project administration, funding acquisition.	Duaa M Bakhshwin
Conceptualization, methodology, Supervision, funding acquisition.	Emad S Aljahdli
Supervision, funding acquisition.	Wid Kattan
Conceptualization, Methodology, Software, validation, formal analysis, investigation, resources, data curation, writing - original draft, writing - review & editing.	Eilaf Majdi Metwalli
Conceptualization, Methodology, Software, validation, formal analysis, investigation, resources, data curation, writing - original draft, writing - review & editing, visualization.	Wafaa Hani Alhashmi
Conceptualization, Methodology, Software, validation, formal analysis, investigation, resources, data curation, writing - original draft, writing - review & editing.	Renad Ahmad ALmutiry
Conceptualization, Methodology, Software, validation, formal analysis, investigation, resources, data curation, writing - original draft, writing - review & editing.	Alya Ali Alrehaili
Conceptualization, Methodology, Software, validation, formal analysis, investigation, resources, data curation, writing - original draft, writing - review & editing.	Asalah Salem Alammari
Conceptualization, Methodology, Software, validation, formal analysis, investigation, resources, data curation, writing - original draft, writing - review & editing.	Manar Oudah Alharbi
